# Interactive Effect of Herbivory and Competition on the Invasive Plant *Mikania micrantha*

**DOI:** 10.1371/journal.pone.0062608

**Published:** 2013-05-30

**Authors:** Junmin Li, Tao Xiao, Qiong Zhang, Ming Dong

**Affiliations:** 1 College of Life and Environmental Sciences, Hangzhou Normal University, Hangzhou, China; 2 Institute of Ecology, Taizhou University, Linhai, China; 3 State Key Laboratory of Vegetation and Environmental Change, Institute of Botany, Chinese Academy of Sciences, Beijing, China; Helmholtz Centre for Environmental Research – UFZ, Germany

## Abstract

A considerable number of host-specific biological control agents fail to control invasive plants in the field, and exploring the mechanism underlying this phenomenon is important and helpful for the management of invasive plants. Herbivory and competition are two of the most common biotic stressors encountered by invasive plants in their recipient communities. We predicted that the antagonistic interactive effect between herbivory and competition would weaken the effect of herbivory on invasive plants and result in the failure of herbivory to control invasive plants. To examine this prediction, thus, we conducted an experiment in which both invasive *Mikania micrantha* and native *Coix lacryma-job* ***i*** were grown together and subjected to herbivory-mimicking defoliation. Both defoliation and competition had significantly negative effects on the growth of the invader. However, the negative effect of 75% respective defoliation on the above- and below-ground biomass of *Mikania micrantha* was alleviated by presence of *Coix lacryma-jobi*. The negative effect of competition on the above- and below-ground biomass was equally compensated at 25%, 50% and 100% defoliation and overcompensated at 75% defoliation. The interactive effect was antagonistic and dependent on the defoliation intensity, with the maximum effect at 75% defoliation. The antagonistic interaction between defoliation and competition appears to be able to release the invader from competition, thus facilitating the invasiveness of *Mikania*, a situation that might make herbivory fail to inhibit the growth of invasive *Mikania* in the invaded community.

## Introduction

Invasive plants pose severe threats to biological diversity and ecosystems [1], and many methods have been used to control invasive plants. Biological control, i.e., using natural enemies to control invasion success, has received much attention [2], [3] and has been highly successfully used to control noxious weeds, such as *Senecio jacobaea* [4] and *Ageratina riparia* [5]. Biological control, being effective and having a low cost and relatively high environmental safety, has been widely accepted [6]. However, many natural enemies have recently been verified as being inefficient in biologically controlling invasive plants in the invaded communities [7], [8], even though the host-specific agents were efficient in pot experiments. Thus, exploring the mechanism underlying this phenomenon would be important and useful in developing future biological controls of invasive species.

It has been noted that the failure of biocontrol might be due to the focus on simple predator-prey relationships and the disregard of more complex interactions in the invaded community [8]. In a natural ecosystem, herbivory and competition are two of the most common biotic stressors that plants encounter [9],[10], and both play important roles in shaping the structure and dynamics of the community [11]; this is true for both the invasive plants and the invaded community [11]. It is well known that both herbivory and competition from native competitors in the invaded community can negatively affect invasive plants and reduce their growth and fitness [12], [13]. Inter-specific competition and herbivory can have synergistic effects on the performance of the attacked invasive host plant [14]–[16] and, as a result, release native neighbours from competition [17], thus limiting invasive success in the invaded community and facilitating the restoration of the native community [18]. However, only few studies have revealed the independent [19] and antagonistic [10], [20] interactive effects of herbivory and competition on invasive plants. We predicted that the antagonistic interactive effect between herbivory and competition could induce the compensatory growth of invasive plants and weaken the effect of herbivory on invasive plants, which would release invasive plants from the naeighbouring competitors and result in the failure of herbivory to control invasive plants. Obviously, an understanding of the interactive effect of herbivory and competition on the performance of invasive plants and the structure and dynamics of the invaded community is important to predict the effectiveness of biological agents on the invasive plants in an invaded community.

*Mikania* (Asteraceae) (hereafter referred to as *Mikania*), a perennial weed native to Central and South America, was introduced into China in ca. 1919 and subsequently became an invader. *Mikania* has caused serious and extensive damage to many Chinese ecosystems, particularly in recent decades [21]. *Mikania* rarely behaves as a weed in its native range because it encounters strong natural enemies in its habitats [22]. Since 1989, herbivores, such as *Liothrips mikania*e, were introduced to Malaysia, India and China but failed in the biological control of *Mikania* [23]; however, the main reason for the failure is still unknown.

*Coix lacryma-jobi* (Poaceae) (hereafter referred to as *Coix*) is a native annual grass, commonly occurring in the communities that are subject to invasion by *Mikania*. We conducted an experiment in which invasive *Mikania* was growing with native *Coix* and was treated with defoliation-mimicking herbivory to examine the interactive effect between herbivory and competition on invasive *Mikania*. We predicted that an antagonistic interaction between herbivory and competition from native species would enhance the performance of the invasive *Mikania* and release it from competition. In particular, we addressed the following questions: 1) Can competition from the native neighbouring *Coix* affect the response of the invasive *Mikania* to defoliation? 2) Can defoliation affect the impact of competition on the invasive *Mikania* and release it from competition? 3) Is the interaction between defoliation and competition antagonistic?

Moreover, the extent to which plants respond to herbivory might be dependent on the intensity of herbivory [24]. Puettmann and Saunders found that the compensatory growth of *Pinus strobes* seedlings varied with the competitive conditions and clipping intensity [24]. Accordingly, we also aimed to address the following question: 4) Does the intensity of defoliation affect the interactive effect? In this study, some physiological traits of invasive *Mikania* were also measured to explore the mechanical responses to the interaction between defoliation and competition.

*Actinote thalia pyrrha* (Fabricius), a natural enemy in the native range of *Mikania*, is currently being introduced to India [25] and China [26], [27] to control *Mikania*. *A. thalia pyrrha* is verified as a potential agent of biological control, as the insect consumes all of the young leaves and stems of *Mikania* [26]. The results of our research could provide information for the management of invasive *Mikania* and also for the application of natural enemies to control invasive *Mikania*.

## Materials and Methods

### Study Site

We conducted our pot experiment in the village of Dengshuiling, southeast of Dongguan City (E 113°31′ −114°15′; N 22°39′−23°09′), Guangdong Province, China. The area has a marine subtropical climate, with a mean annual precipitation of 1819.9 mm, mean annual temperature of 23.1°C and mean annual sunshine time of 1873.7 hr. The zonal vegetation is subtropical evergreen broadleaved forest codominated by *Dactyloctenium aegyptium, Paederia scandens* and *Pharbitis nil. Mikania* began to invade this area in the early 1990 s and spread extensively in shrublands and old fields.

### Experimental Design and Measurements

Invasive *Mikania* was collected from the fields surrounding Dengshuiling and then propagated using cuttings. The site is located in an open and abandoned field, and no specific permits were required for the described field studies. Native *Coix* was germinated from seeds that were purchased from Shandong Heze Chinese Medicine Institute. We filled our experimental pots (3 L) with field-collected red clay soil mixed with sand (3∶1).

Artificial defoliation has been employed extensively as a method of simulating herbivore attack [12],[28]–[30] and has recently been used to simulate biological agents to control invasive plants [20], [31], [32]. Although artificial defoliation does not always elicit the same results as true herbivory, it can allow researchers to control the amount of defoliation precisely [20]. We used defoliation to mimic the herbivory that plants are likely to encounter in nature. A factorial combination of defoliation intensities (0%, 25%, 50%, 75% or 100%) and competition (with or without) were applied to treat invasive *Mikania*. A total of 10 treatments were used in this experiment, and 5 replicates were used for each treatment, amounting to 50 pots. For the experiment without competition, an individual *Mikania* plant was transplanted into each pot; for the competition treatment, an individual *Mikania* plant and one *Coix* plant of similar size were transplanted together into each pot with a distance of 15 cm between them. The pots were irrigated with tap water twice daily and fertilised with 50% Hoagland's nutrient solution once per week [33]. Bamboo sticks (1 m long) were inserted into the soil near *Mikania* to allow the plant to climb. Three weeks after transplantation, *Mikania* plants of similar size were chosen for defoliation. Herbivory by *A. thalia pyrrha* on *Mikania* can remove all of the leaves [26]. To simulate a realistic intensity of herbivory, five intensities were included in this experiment: (1) 0% defoliation, (2) 25% defoliation, (3) 50% defoliation, (4) 75% defoliation, and (5) 100% defoliation. These treatments constituted removing 0%∼45% of the total above-ground biomass at the time of clipping to simulate zero to moderate aboveground herbivory [34]. The defoliation of *Mikania* was performed by removing each leaf with scissors, leaving the petiole attached to the stem.

After four weeks from the date of the first defoliation, a second defoliation at different levels was conducted on the newly emerging leaves. The physiological responses of plants to defoliation have received considerable attention and are considered a potential mechanism of the compensatory growth response to defoliation [35]. After three weeks from the date of the second defoliation, the net photosynthetic rate (P_n_), transpiration rate (*E*) and leaf photosynthetically active radiation (PAR) of the *Mikania* plants were measured using a portable photosynthesis and transpiration system (LCA-4, Analytical Development Co. Ltd, Hoddesdon, UK) on the terminal leaflet of the third mature leaf from the top of the plant. The measurements were performed between 9∶00 and 11∶00 am under light intensity of 1400 μmol m^−2^ s^−1^, leaf temperature of 30°C, CO_2_ concentration of 350 ppm and relative moisture of 55%. The light use efficiency was calculated as *P*_n_/PAR [36], and the water use efficiency as *P*_n_/*E* [37]. The harvested plants were then separated into shoots and roots and dried for 48 h at 80°C to determine the final total biomass.

### Data Analyses

To investigate the effects of herbivory and competition on the growth of *Mikania* in more detail, we calculated four response indices for the above-ground biomass of *Mikania*: defoliation responses (DR = with defoliation/without defoliation) and competition responses (CR = with competition/without competition [10]. This calculation is based on a null model that competition and herbivory do not interact and respond multiplicatively on a linear scale. If DR or CR = 1, there would be no effect of competition or herbivory on plant growth. If DR or CR <1, there would be a negative effect; If DR or CR>1, there would be a positive effect. We also calculated TR_pred_ (DR × CR) to indicate the simple multiplicative effects of competition and herbivory together on plant growth and TR_true_ (with defoliation and competition/without defoliation and competition) to indicate the observed combined effect of both competition and herbivory [10]. If TR_pred_ > TR_true_, there would be a synergistic interaction between competition and herbivory; If TR_pred_ < TR_true_, there would be an antagonistic interaction. If TR_pred_ = TR_true_, there would be no interaction.

Two-way analysis of variances (ANOVAs) were used to analyse the factorial effect of the defoliation intensities (0%, 25%, 50%, 75% or 100%) and competition (with or without) on the growth of the invasive plant, with defoliation and competition as the main factors. One-way ANOVAs were used to analyse the effect of defoliation on the growth of the invasive plant with or without the competitor. In the ANOVAs, the response indices were log-transformed and the other indices were log-transformed only when the assumption of homoscedasticity of the indices was not met. The homogeneity of the variance was evaluated using Levene's test. Statistical significance was taken at p<0.05.

## Results

### Effect of Competition on *Mikania* Responses to Defoliation

**Figure 1:**
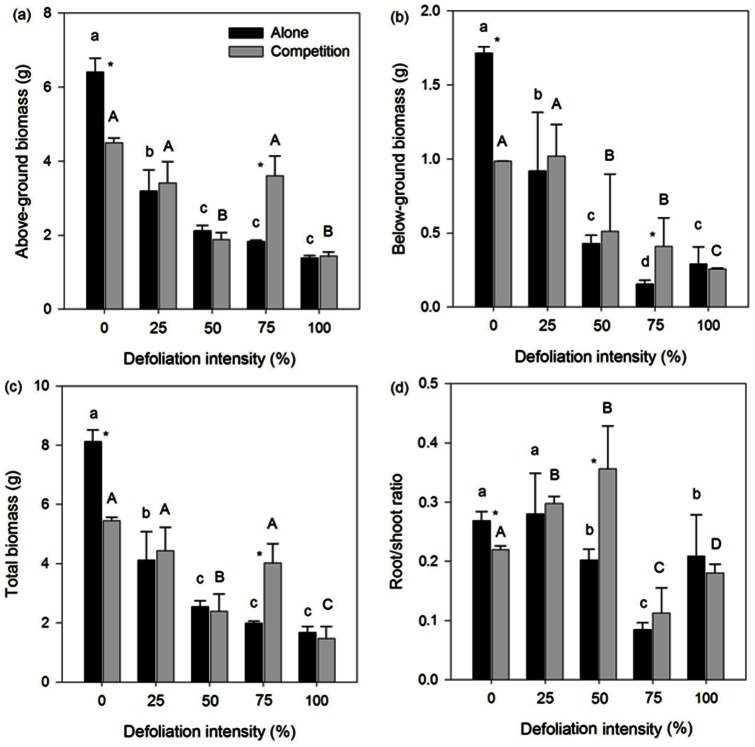
Effect of defoliation and competition on the above-ground (a), below-ground (b), total biomass (c) and root/shoot ratio (d) of invasive *Mikania micrantha.* Values are means ±standard deviation. The different lowercase and uppercase letters indicate significant differences (*p*<0.05) among the defoliation intensities of invasive *Mikania micrantha* growing with and without a native competitor, respectively. * indicates a significant difference (*p*<0.05) between the treatments with or without the competitor.

**Figure 2:**
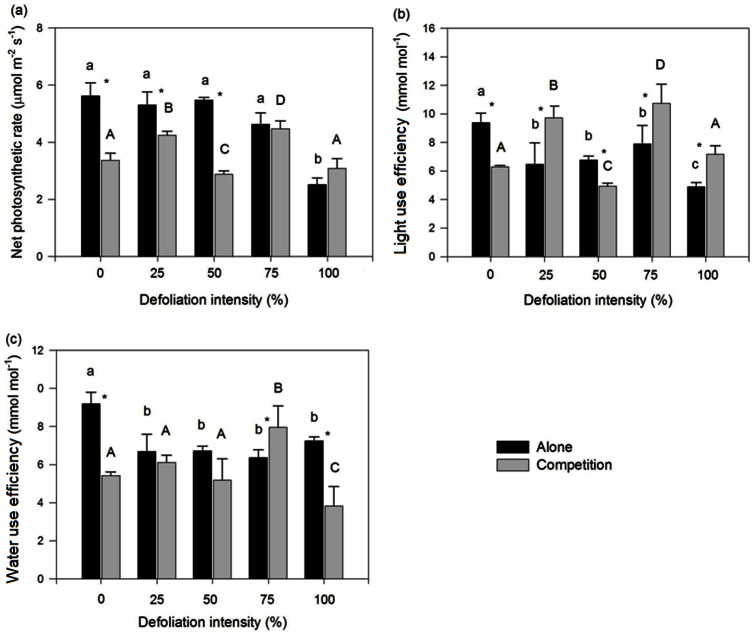
Effect of defoliation and competition on the net photosynthetic rate (a), light use efficiency (b) and water use efficiency (c) of invasive *Mikania micrantha*. Values are means ±standard deviation. The different lowercase and uppercase letters indicate significant differences (*p<0.05*) among the different intensities of defoliation of invasive *Mikania* micrantha growing with and without native competitor, respectively. * indicates significant differences (*p*<0.05) between the treatments with or without competitor.

Defoliation significantly decreased the above- and below-ground and total biomass of *Mikania* growing alone, whereas 25% and 75% defoliation had no significant effect on the growth of *Mikania* growing with native *Coix* except for the below-ground biomass (Fig. 1). Defoliation intensities from 50% to 100% significantly reduced the root/shoot ratio of *Mikania* when growing alone, whereas 25% and 50% defoliation significantly increased the ratio of *Mikania* growing with native *Coix* (Fig. 1). Defoliation significantly decreased the light use efficiency and water use efficiency, yet only 100% defoliation significantly decreased the net photosynthetic rate of *Mikania* growing alone (Fig. 2). Defoliation intensities of 25% and 75% significantly increased the net photosynthetic rate and light use efficiency while 75% defoliation significantly increased the water use efficiency of *Mikania* growing with native *Coix* (Fig. 2). The two-way ANOVAs results showed that defoliation had a significant effect on all of the growth and physiological traits of *Mikania* (Table 1).

**Table 1:** *F* values of the two-way ANOVAs for testing the effects of defoliation (different intensities) and competition (with or without) on the growth and physiological traits of *Mikania* micrantha.

Traits	Competition	Defoliation	Competition × Defoliation
Above-ground biomass	0.033	133.707***	23.486***
Below-ground biomass	0.868	38.135***	6.510*
Total biomass	0.287	103.219***	18.381***
Root/shoot ratio	3.142	25.187**	6.724**
Net photosynthetic rate	103.013**	52.913***	29.446***
Water use efficiency	37.650**	7.529***	16.473***
Light use efficiency	4.807***	19.137***	16.161***

Figures in bold are significant at *p*<0.05; Significance levels: *p<0.05, **p<0.01, ***p<0.001.

**Figure 3:**
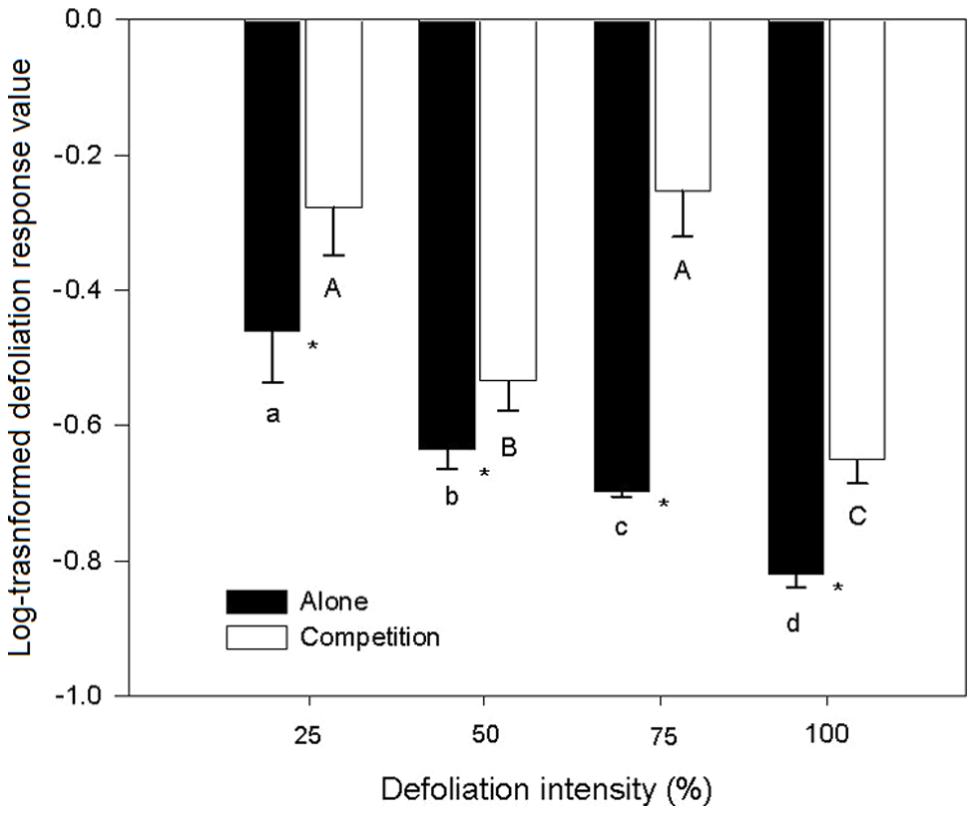
Log-transformed response values of *Mikania micrantha* with and without competition to defoliation intensities. Values are means±standard deviation. The different lowercase and uppercase letters indicate significant differences (*p*<0.05) among the defoliation intensities of invasive *Mikania* micrantha growing with and without native competitor, respectively. * indicates significant differences (*p*<0.05) between the treatments with or without competitor.

In terms of the above-ground biomass, the defoliation response values of *Mikania* were all less than 0 and decreased with increasing defoliation intensities (Fig. 3), indicating a negative effect of defoliation on *Mikania*, regardless of the presence of competition: the more leaves that were removed, the more the above-ground biomass was decreased. However, the response values to the defoliation intensity of *Mikania* growing with native *Coix* were all significantly higher than those of *Mikania* growing alone, particularly at 75% defoliation (Fig. 3), indicating a compensatory growth of *Mikania* to defoliation was induced by the growth of native *Coix*.

### Effect of Defoliation on *Mikania* Responses to Competition

Competition significantly decreased the above- and below-ground and total biomass of *Mikania* at 0% defoliation (Fig. 1). When *Mikania* was treated with 25%, 50% and 100% defoliation, competition had no effect on its growth; in contrast, competition significantly increased growth when *Mikania* was treated with 75% defoliation (Fig. 1). Competition significantly decreased the root/shoot ratio at 0% defoliation and significantly increased the root/shoot ratio at 50% defoliation but had no effect at 25%, 75% and 100% defoliation (Fig. 1). Competition significantly decreased the net photosynthetic rate, light use efficiency and water use efficiency at 0% defoliation, whereas 75% defoliation resulted in a similar net photosynthetic rate and a greater water use efficiency; 25%, 75% and 100% defoliation increased the light use efficiency, with a statistical significance at 100% defoliation (Fig. 2). The two-way ANOVAs results showed that competition had a significant effect on the root/shoot ratio, net photosynthetic rate, light use efficiency and water use efficiency (Table 1).

**Figure 4:**
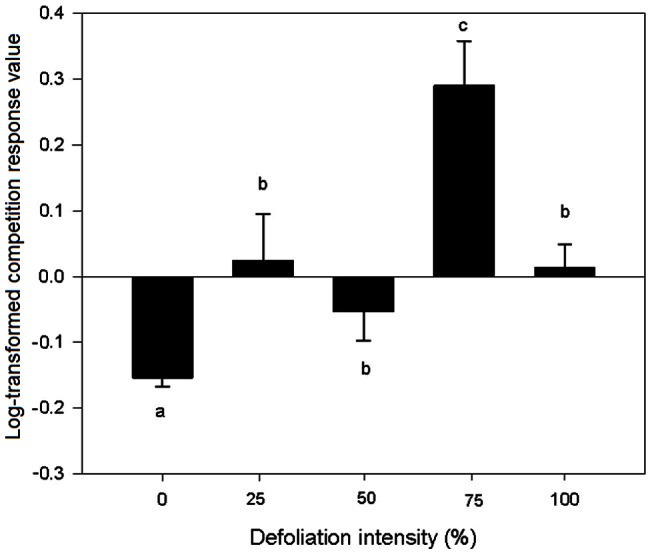
Log-transformed response values of *Mikania micrantha* to competition at different intensities of defoliation. **Values are means±standard deviation.** Different lower case letters indicate significant differences between the defoliation intensities at *p*<0.05.

Based on the above-ground biomass, the competition response values of *Mikania* at 0% and 50% defoliation were less than 0, whereas those at 25%, 75% and 100% defoliation were more than 0, indicating that competition had a negative effect on the growth of *Mikania* at 0% and 50% defoliation but had a positive effect on the growth of *Mikania* at 25%, 75% and 100% defoliation. The competition response values of *Mikania* at different defoliation intensities were higher than those without defoliation, particularly at 75% (Fig. 4), indicating that defoliation could alleviate the negative effect of competition on the growth of *Mikania*.

### Interactive Effect of Competition and Defoliation on *Mikania*

**Figure 5:**
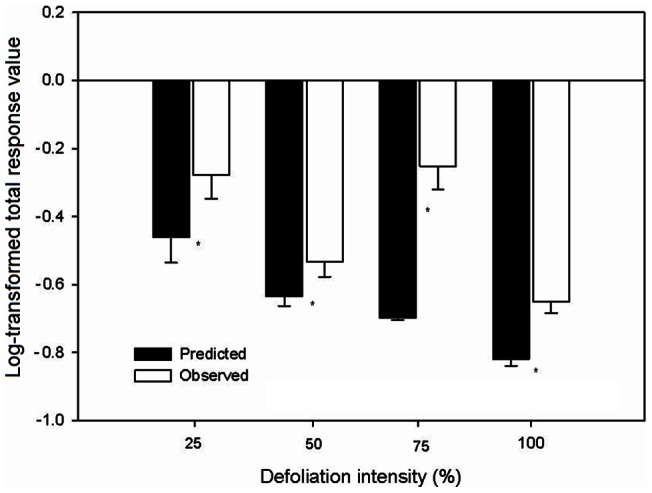
Log-transformed total predicted and observed response values to defoliation and competition of invasive *Mikania micrantha* defoliated at different intensities. Values are means±standard deviation. The different lowercase letters indicate a significant difference between the defoliation intensities at *p*<0.05. * indicates significant differences (*p*<0.05) between the treatments with or without competitor.

Both competition and defoliation significantly reduced the growth of *Mikania* compared to the plants of the species grown without competition and defoliation (Fig. 1). The TP_true_ values were all significantly higher than the TP_pred_ values, regardless of the intensity with which *Mikania* was defoliated, indicating an antagonistic interactive effect between competition and defoliation on the growth of *Mikania* (Fig. 5). The two-way ANOVAs results showed that the defoliation × competition interaction had a significant effect on all of the growth and physiological traits of *Mikania* (Table 1).

## Discussion

Both competition and herbivory by native species could affect the invasiveness of introduced species and often limit the success of invasive species in a recipient community [18]. However, the compensatory growth responses of plants after herbivory damage can alleviate the potential deleterious effects of herbivory and can have a positive impact on the fitness of plants [38], intensifying the negative impact on the native neighbour and releasing the invasive species from competition [39]. Walling and Zabinski have found that the competitive ability of invasive *Centaurea maculosa* to outgrow native plants was intensified by the compensatory growth produced by defoliation, which resulted in a greater capture of resources [31]. In our study, just as we predicted, the effect of the interaction between competition and defoliation on the growth of *Mikania* was less than their individual effects, indicating an antagonism. Similar antagonistic effects have also been found in invasive *Centaurea melitensis* [20], *Centaurea solstitialis* [34] and *Poa annua* [10]. In the present study, the antagonistic interactive effect of defoliation and competition from native *Coix* on invasive *Mikania* and the consequent compensatory growth of *Mikania* might be one of the possible mechanisms why host-specific biological control agents could not successfully control invasive plants in an invaded community.

It has been commonly verified that plants may compensate for tissue losses due to defoliation, resulting in increased growth relative to non-defoliated plants [30], [40]. Different from these conclusions, in this study, defoliation had a negative effect on the growth of invasive *Mikania* growing alone: growth declined with increasing defoliation intensities. However, the negative effect of defoliation may be modified by competition. The response values to different defoliation intensities tested on *Mikania* growing with native *Coix* were all significantly higher than those of *Mikania* growing alone, indicating a compensatory growth of *Mikania* induced by competition in response to defoliation, particularly at 75% defoliation. This result indicates that native *Coix* could help invasive *Mikania* be more vigorous after defoliation.

Although the mechanism underling the compensatory growth of *Mikania* that is induced by the competition is unknown, the underground network between the roots of invasive *Mikania* and native *Coix* mediated by mycorrhizae might be a possible mechanism. Although it is still unknown why defoliation can induce a potential transfer of nutrients between a plant and a neighbouring plant, evidence using stable isotopes verified that defoliation could change the underground nitrogen flow [41] and that carbon could be transferred via mycorrhizae from native neighbouring plants to the invasive plant [42]. Native *Coix* is a mycorrhizal plant [43], and the soil in the *Mikania* community is rich in fungi [44]. It has also been verified that native neighbours are capable of enhancing compensatory growth of invasive plants to defoliation in the presence of soil fungi [20], [34]. Further atention should be paid to the underground mechanism.

The successfully invasive plants are always strong competitors of the native plant species, however, native plants has been verified as a major force in the resistance of exotic invasions [3], [45]. In this study, competition from native *Coix* did significantly decrease the growth of invasive *Mikania* because of the limited resources. However, the negative effect of competition on the growth of *Mikania* may be modified by defoliation. The response values of *Mikania* to competition increased at each defoliation intensity, indicating a release from native competitor *Coix* induced by defoliation, particularly at 75% defoliation. The release of *Mikania* from competition that can be induced by defoliation could increase the number of invasive plants and allow the domination of niche spaces to the detriment of native species [46], perhaps facilitating the invasiveness of *Mikania* and helping to shape the structure and dynamics of the invaded communities.

Plants have the ability to (at least partially) compensate for herbivory only above a certain threshold level of damage [29], and this threshold can differ among plant species. Yu et al. found that invasive *Alternanthera philoxeroides* can only rapidly recover from 50% defoliation [47]. Similarly, in the present study, when the native *Coix* was present, 75% defoliation induced the compensatory growth of invasive *Mikania*. Many morphological and physiological mechanisms have been proposed to explain the compensatory growth that follows herbivory or defoliation [30], such as the increased allocation of substrates from the roots to shoots [48] and the increased photosynthetic rate of the regrowing tissue [49]. In our study, 75% defoliation decreased the root/shoot ratio and significantly increased net photosynthetic rate, light use efficiency and water use efficiency. The resources stored in the roots were shifted to the shoots, significantly reducing the root/shoot ratio [50]. Barton found that *Plantago lanceolata* (Plantaginaceae) seedlings were plastic in their resource allocation between the shoots and roots, resulting in compensatory growth [50]. This type of strong compensatory growth due to phenotypic plasticity and the physiological acclimation of invasive *Mikania* was maximised at 75% defoliation.

Although artificial defoliation has been widely used to mimic the effect of truly herbivory on plants [12], [28]–[30], [51], there are undeniably significant differences between defoliation and herbivory [52]. Artificial defoliation can only mimic the effect of the loss of leaf area which decreased the ability of plants to intercept light [53] but not the effect in responding to the physiological and chemical interactions (e.g., due to nutrient supply) between herbivores and plants. In spite of some pitfalls, artificial defoliation has been used more often in herbivory research than real herbivores for easily and precisely controlling, targeted effect and efficient experimental designs [53]. And there were only a few cases (as low as 3%) with the outcomes where artificial and natural damage had opposite effects on plants. The biological control agent of *Mikania* are found to consume all of the young leaves and stems of *Mikania* [26], so the defoliation can at least partially mimic the effect of the loss of leaf area caused by the biological control agent.

In conclusion, our results suggest that natural herbivory might not necessarily be safely used as a potential agent to control invasive *Mikania* in the field because of the induced compensatory growth of *Mikania* by native *Coix*. Further studies should consider the interactions at the intertrophic and multitrophic levels in invaded communities as well as among more factors including, e.g., nutrient supply which seems difficult to investigate with simulated herbivore, whereby the ecological risk of the releasing of the biological control agents can be comprehensively evaluated.
